# A Mendelian randomization analysis identifies causal association between sarcopenia and gastroesophageal reflux disease

**DOI:** 10.18632/aging.205627

**Published:** 2024-03-05

**Authors:** Renwang Hu, Can Liu, Dan Li

**Affiliations:** 1 Department of Gastrointestinal Surgery, Henan Provincial People’s Hospital, Zhengzhou, Henan, China; 2 Department of Gastrointestinal Surgery, Zhengzhou University People’s Hospital, Zhengzhou, Henan, China; 3 Department of Radiology, Henan Provincial People’s Hospital, Zhengzhou, Henan, China

**Keywords:** gastroesophageal reflux disease, sarcopenia, causal association, Mendelian randomization

## Abstract

The incidence of gastroesophageal reflux disease (GERD) is increasing with the advancement of world population aging, affecting the population health worldwide. Recently, there were several researches to suggest the association between GERD and sarcopenia, but evidence supporting the causal effect was absent. The purpose of this study is to determine the causal relationship between GERD and sarcopenia through a Mendelian randomization (MR) study. We conducted an MR analysis by using summary-level data of genome-wide association studies (GWASs) in the European population. The inverse variance weighted (IVW) method was used as the primary analytical method for evaluating causality. In addition, four other MR methods were performed to supplement the IVW results. We also used the Mendelian randomization pleiotropy residual sum and outlier (MR-PRESSO) and the multivariable Mendelian randomization (MVMR) to validate the robustness of our results. IVW analysis revealed a causally positive correlation between low hand grip strength (OR = 1.2358, 95% C.I.: 1.0521-1.4514, P = 0.0099), decreased walking pace (OR = 0.1181, 95% C.I.: 0.0838-0.1666, P = 4×10^-34^), and decreased appendicular lean mass (ALM) (OR = 0.8612, 95% C.I.: 0.8263-0.8975, P = 1×10^-12^) and GERD. MR-PRESSO and MVMR analysis confirmed the association evidence. In conclusion, this MR analysis supported the causal association between sarcopenia-related traits and GERD.

## INTRODUCTION

Gastroesophageal reflux disease (GERD) is defined as recurrent, troublesome heartburn and reflux symptoms or GERD-specific complications [1–3]. In recent years, with the intensification of global aging and changes in lifestyle worldwide, the incidence of GERD has increased [4, 5], contributing to the overall global burden of disease [3, 5, 6].

Sarcopenia was first defined by Irwin H. Rosenberg in 1988 to describe age-related loss of skeletal muscle quantity and quality [7]. In 2010, the European Working Group on Sarcopenia in Older People (EWGSOP) clarified the definition of sarcopenia: sarcopenia is a syndrome characterized by progressive and comprehensive loss of skeletal muscle mass and muscle strength, accompanied by the risk of adverse consequences such as physical disability, poor quality of life, and death [8]. Currently, the role of skeletal muscle quality and quantity in clinical outcomes and disease prevention has received increasing attention [9, 10].

Observational studies indicated that the presence of sarcopenia was positively associated with GERD [11, 12], but causal effect evidence was still absent for this conclusion. Mendelian Randomization (MR) study is an innovative research method that uses genetic variables (single nucleotide polymorphisms, SNPs) as instrumental variables (IVs) to investigate causal effects between exposures and outcomes, avoiding the effects of confounding variables and reverse causal relationships [13, 14].

This present study aimed to determine the causal associations between GERD and sarcopenia through bidirectional MR analysis, to provide evidence for clinical diagnosis, treatment, and disease prevention.

## RESULTS

**Figure 1:**
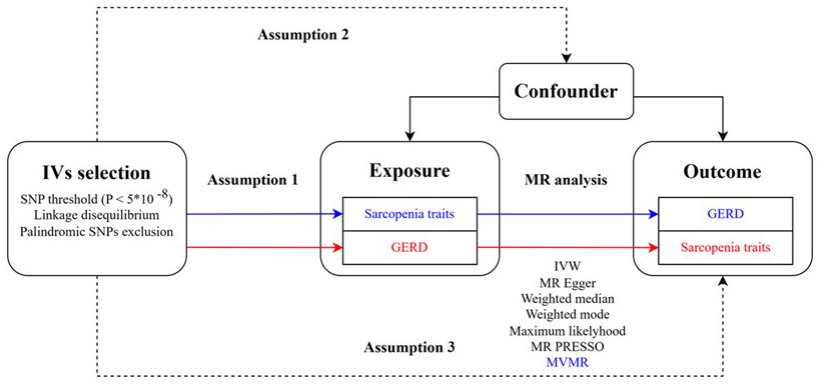
**The overall flow chart of the MR study.** Assumption 1 is that the genetic variants used as instrumental variables should be robustly associated with the exposure; assumption 2 is that the used genetic variants should not be associated with any confounders; and the assumption 3 is that the used instrumental variables should affect the risk of the outcome solely through the exposure, not via other pathways. GERD, gastroesophageal reflux disease; IV, instrumental variable; SNP, single nucleotide polymorphism; MR, Mendelian randomization; MR-PRESSO, Mendelian randomization pleiotropy residual sum and outlier; IVW, inverse variance weighted; MVMR, multivariable Mendelian randomization.

The overall flow chart for this MR study is depicted in Figure 1. Firstly, we set sarcopenia-related traits as exposure factors and GERD as the outcome variable for MR analysis. In the screening of IVs, we obtained 10, 46, and 404 SNPs strongly correlated to exposure factors at the genome-wide threshold (P < 5 × 10^-8^) in low hand grip strength, walking pace, and ALM, respectively. F-statistics of all these selected SNPs were greater than 10 (Supplementary Table 2). The results of the heterogeneity test and horizontal pleiotropy test are shown in Supplementary Table 3. Cochran’s Q test suggested that heterogeneity was present among all SNP groups selected for analysis (P < 0.05); thus, a random effects IVW model was used in subsequent analysis. In the horizontal pleiotropy test, SNPs associated with low grip strength suffered from horizontal pleiotropy (intercept = -0.0523, P = 0.0102), while no significant horizontal pleiotropy was found in the other two tests (P = 0.3651 and P = 0.0656).

**Figure 2:**
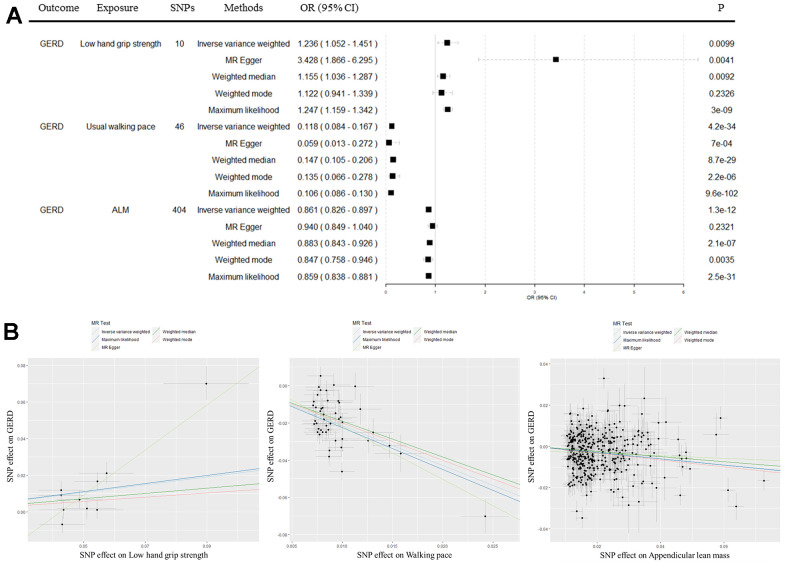
**Causal effects of sarcopenia traits on gastroesophageal reflux disease.** (**A**) Forest plot for causal effects of sarcopenia traits on gastroesophageal reflux disease. (**B**) Scatter plot for causal effects of low hand grip strength, usual walking pace, and appendicular lean mass on gastroesophageal reflux disease, respectively. Analyses were performed by using the Inverse variance weighted, MR Egger, Weighted median, Weighted mode, and Maximum likelihood methods. The slope of each line corresponds to the estimated MR effect per method. GERD, gastroesophageal reflux disease; ALM, appendicular lean mass; MR, Mendelian randomization; SNP, single nucleotide polymorphism.

The random effects IVW results suggested that there were causal effects of the sarcopenia-related traits on GERD (Figure 2A). Low grip strength (OR = 1.2358, 95% C.I.: 1.0521-1.4514, P = 0.0099), decreased walking pace (OR = 0.1181, 95% C.I.: 0.0838-0.1666, P = 4×10^-34^), and decreased ALM (OR = 0.8612, 95% C.I.: 0.8263-0.8975, P = 1×10^-12^) could lead to an increased risk of GERD. In addition, other MR analyses suggested similar causal effects (Supplementary Table 5 and Figure 2B).

We then identified outlier SNPs using the MR-PRESSO method (Supplementary Table 4). After excluding outlier SNPs, we conducted MR analysis again, and IVW results revealed that low grip strength (OR = 1.1955, 95% C.I.: 1.0852-1.3170, P = 0.0003), decreased walking pace (OR = 0.1291, 95% C.I.: 0.0958-0.1740, P = 4×10^-41^), and decreased ALM (OR = 0.8742, 95% C.I.: 0.8430-0.9064, P = 4×10^-13^) would increase the risk of GERD, which were generally consistent with the results before correction (Supplementary Table 5). MVMR suggested that after adjusting for BMI, alcohol intake frequency and smoking initiation, low hand grip strength (OR = 1.1213, 95% C.I.: 1.0487-1.1988, P = 0.0008), walking pace (OR = 0.2924, 95% C.I.: 0.2146-0.3983, P = 6×10^-15^) and ALM (OR = 0.8616, 95% C.I.: 0.8271-0.8975, P = 9×10^-13^). After additionally adjusting for coffee consumption and type 2 diabetes, the results did not substantially change (Table 1), validating the robustness of the univariable MR results.

**Table 1:** Multivariable Mendelian randomization results of the causal effect of sarcopenia traits on gastroesophageal reflux disease after adjusting for confounding variables.

**Exposure**	**OR**	**95% C.I.**		**P**
		**Lower**	**Upper**	
**MVMR 1**				
Low hand grip strength	1.121	1.049	1.199	8.0×10^-04^
Usual walking pace	0.292	0.215	0.398	6.3×10^-15^
Appendicular lean mass	0.862	0.827	0.898	9.2×10^-13^
**MVMR 2**				
Low hand grip strength	1.118	1.040	1.202	2.6×10^-03^
Usual walking pace	0.263	0.186	0.370	2.1×10^-14^
Appendicular lean mass	0.830	0.785	0.878	5.8×10^-11^
**MVMR 3**				
Low hand grip strength	1.117	1.040	1.199	2.3×10^-03^
Usual walking pace	0.275	0.196	0.384	4.8×10^-14^
Appendicular lean mass	0.870	0.833	0.909	3.1×10^-10^

OR, odds ratio; C.I., confidence interval; MVMR, Multivariable Mendelian randomization;

MVMR 1, adjusted for body mass index, alcohol consumption and smoking initiation;

MVMR 2, adjusted for body mass index, alcohol consumption, smoking initiation and type 2 diabetes;

MVMR 3, adjusted for body mass index, alcohol consumption, smoking initiation, type 2 diabetes and coffee consumption.

We then set genetically predicted GERD as an exposure factor and sarcopenia-related traits as outcome variables for MR analysis to explore potential causal effects. We obtained 77 SNPs strongly related to exposure at the genome-wide threshold (P < 5 × 10^-8^). All of these selected SNPs were valid (Supplementary Table 2). Heterogeneity was present in all of these analyzed SNP groups, while no significant horizontal pleiotropy was found (Supplementary Table 3).

The random effects IVW results suggested a causal effect of genetically predicted GERD and on sarcopenia-related traits (Supplementary Table 6). Other methods of MR analyses suggested similar results (Supplementary Table 6). Subsequently, after excluding outlier SNPs (Supplementary Table 4), the results of MR analysis were proved robust (Supplementary Table 6).

## DISCUSSION

In this study, we investigated the potential causal association between sarcopenia and GERD by using two-sample, bidirectional MR analysis. The main finding was that sarcopenia-related traits have causal effects on GERD. This current study supplemented the previous researches on the association between sarcopenia and GERD, and proposed that genetically predicted GERD might have an impact on sarcopenia. To our knowledge, this is the first study that uses GWAS summary-level data and MR analysis to explore the causal association between GERD and sarcopenia.

MR analysis is a major advantage of this study. Randomized controlled trials (RCTs) are commonly used to determine the causal relationship of exposures on outcomes, while the implementation of RCTs often means significant time and financial costs. MR studies investigate causal relationships by using genetic instrumental variables related to exposures and outcomes, avoiding the effects of confounding factors and reverse causality, and are an effective and efficient alternative research method for RCTs [15]. Our IVW-MR analysis revealed a causal relationship between the sarcopenia-related traits and GERD (Figure 2 and Supplementary Table 5). In addition, the results from the other four MR methods are generally consistent with the IVW results. Although the findings of some MR-Egger and weighted mode results were not statistically significant (P > 0.0167), the estimated effects were still in the same direction, which proved the robustness of the research results. We also repeated MR analysis after removing outliers SNPs and conducted MVMR, the results were generally consistent with those before correction (Table 1 and Supplementary Table 5).

The risk of GERD is influenced by many factors, such as age, obesity, smoking, alcohol and coffee consumption, and so on [10, 16]. By adjusting those confounding variables in MVMR, we confirmed that sarcopenia was also an important risk factor for GERD. Imagama et al. conducted a prospective cohort study on 178 healthy samples from the East Asian population. After five years of follow-up, 36.8% of the samples who developed GERD had sarcopenia, while only 10% of the control group had sarcopenia (P < 0.05). Therefore, they proposed that sarcopenia is an important risk factor for GERD [11]. Another study from South Korea [12] analyzed 8218 samples and found that sarcopenia was an independent predictor of GERD (OR = 1.170, CI: 1.016-1.346, P = 0.029). This study further confirmed the reliability of this conclusion through MR analysis for the European population. We thought that the possible pathological mechanism behind this was the insufficient strength of the muscles located at the lower esophageal, which may lead to the reflux of stomach contents into the esophagus. In addition, the weakness of the lumbar muscles may result in deformation or position changes of the spine, causing changes in abdominal pressure, and subsequently leading to changes in pressure in the stomach and lower esophagus, which results in GERD [17].

Inconsistent with previous observational studies, this study also suggested that GERD may reversely affect the emergence of sarcopenia. To our knowledge, there are currently no reports of GERD causing muscle loss, the detailed mechanistic linkage between them remains largely unclear and needs further researches. We thought that the lack of collagen and the effect of inflammatory factors might play important roles in it [18–20]. Furthermore, the appearance of GERD symptoms limits the patient’s physical activity ability [21], and abdominal symptoms affect the patient’s nutritional intake to some extent [22]. Muscle wasting and insufficient nutrition could lead to a loss of skeletal muscle mass.

Our research findings provide new insights for clinical diagnosis and treatment: in patients diagnosed with sarcopenia, it is necessary to be alert about the possibility of GERD. The exercise of muscle strength in the elderly may prevent the occurrence of GERD to some extent, and even alleviate the symptoms of GERD [16, 23]. Similarly, in patients clinically diagnosed with GERD, we may need to pay attention to changes in patients’ muscle mass and take timely intervention measures to avoid the condition worsening and vicious cycle [20].

Our research had several strengths. MR analysis is less susceptible to the influence of unknown confounding factors and reverse causality which are the disadvantages of observational studies. The evaluation of outliers, extensive sensitivity analyses and MVMR that adjusted multiple confounders increased the robustness of the results, and thus strengthened the evidence we found. Although there was sample overlap, according to our calculation results (data not shown), this would not bring significant bias to the results. However, there were still some limitations to mention. First, the data used in this study were all from European populations, making it difficult to extend the conclusions to other populations. Second, due to the application of GWAS summary-level data, it was not possible to perform stratified analysis based on parameters like age and gender. In addition, we used the sarcopenia-related traits in our MR analysis, which may not completely replace the occurrence of sarcopenia as EWGSOP proposed. Finally, although the pleiotropy test and MR-PRESSO method were conducted to prevent confounding by pleiotropy, residual bias can hardly be avoided, as it is a recognized shortcoming of MR analysis.

In conclusion, we confirmed the causal association between GERD and sarcopenia through MR analysis. The relevant mechanisms need further exploration in the future.

## MATERIALS AND METHODS

### Study design

In this study, we conducted a two-sample MR study design, using different Genome-wide Association Studies (GWASs) summary level datasets to investigate the causal relationship between sarcopenia-related traits and gastroesophageal reflux disease in the European population. The flowchart of this MR study is shown in Figure 1. We mainly analyzed whether sarcopenia-related traits have a causal effect on gastroesophageal reflux disease, and validated our results by Mendelian randomization pleiotropy residual sum and outlier (MR-PRESSO) [24] method and multivariable Mendelian randomization (MVMR). Additionally, we reversely explored whether genetically predicted GERD has a causal effect on sarcopenia-related traits.

### Data source

The GWAS summary level data of GERD was extracted from a recently published genome-wide association meta-analysis study [25], which included 129080 European GERD patients and 473524 healthy controls.

We obtained the data of the three most commonly used traits to determine the presence of sarcopenia [26–28]: grip strength, walking pace, and appendicular lean mass (ALM). Grip strength is highly correlated with the full body muscle quality, so it can be used as a reliable substitute for measuring full body strength [29]. Walking paces is considered a fast and safe method for detecting muscle loss and is widely used in clinical practice [29]. ALM is a commonly used muscle mass approximator in sarcopenia researches and is widely used in the diagnostic criteria of EWGSOP [29] and the Asian Working Group for Sarcopenia (AWGS) [30]. The GWAS data for grip strength, walking pace, and ALM were obtained from the United Kingdom Biobank (UKB) [31]. In brief, the UKB is a large prospective cohort study, which includes in-depth information for the genetic composition and health of over 500000 individuals aged between 40 and 69 who participated in the study in the United Kingdom [31].

GWAS summary statistics of confounding factors including body mass index (BMI), smoking initiation, alcohol intake frequency, coffee intake, and type 2 diabetes were derived from Genetic Investigation of Anthropometric Traits (GIANT) consortium, GWAS and Sequencing Consortium of Alcohol and Nicotine use (GSCAN), Medical Research Council Integrative Epidemiology Unit (MRC-IEU) and a GWAS conducted by Xue et al., respectively [32–35].

Supplementary Table 1 summarizes the data sources for this study. All the data presented in Supplementary Table 1 have been approved by the relevant review boards, and informed consent was obtained from all the participants involved.

### Instrumental variables selection

We selected instrumental variables based on three generally recognized assumptions [36]: (1) The IVs need to be strongly correlated with exposure factors; (2) The IVs are not associated with confounding factors; (3) The IVs are solely related to the outcomes through exposures without a direct association with outcomes.

We first selected instrumental variables closely related to exposure factors at the genome-wide significance threshold (P < 5 × 10^-8^). We then estimated the linkage disequilibrium among SNPs using 1000 Genomes European panel as the reference population. Independent SNPs (i.e., SNPs without linkage disequilibrium, defined by r^2^ < 0.001 and clumping window size > 10000 kb) were used as instrumental variables.

In addition, to ensure that the potential instrumental variables have sufficient power to detect the causal effect of exposure on the outcomes, we calculated the F-statistic of the potential IVs using the formula F = R^2^ (n-k-1) / k (1-R^2^) [37] (n represents the sample size, k represents the number of instrumental variables, R^2^ was calculated by the formula R^2^ = 2 × EAF_i_ × (1 - EAF_i_) ×β_i_^2^, EAF_i_ is the effect allele frequency, and β_i_ is the estimated genetic effect on exposure [38]. The IVs with F-statistic > 10 were considered to have sufficient robust estimation power to determine causal effects and were retained. Since the exact biological functions of many genetic variations are still unclear, we also used the MR-PRESSO method to identify and remove pleiotropic SNPs [24].

### Statistical analysis

The inverse variance weighted Mendelian randomization (IVW-MR) method is the primary analytical method used to estimate the causal associations between GERD and sarcopenia-related traits (grip strength, walking pace, and ALM), which is an extension of the Wald ratio estimator based on the meta-analysis principles [39]. In this study, the Cochran’s Q test was used to determine the heterogeneity among the selected SNPs. If significant heterogeneity was observed (P < 0.05), the random effects IVW model was applied; Otherwise, the fixed-effect IVW model was adopted. We also used other methods including MR Egger [40], weighted median [41], maximum likelihood [42], and weighted mode [43] for sensitivity analysis to supplement and validate the results of IVW-MR analysis. The MR-Egger intercept test [44] was used to monitor whether MR analysis is affected by horizontal pleiotropy. Subsequently, we removed the discovered pleiotropic SNPs determined by MR-PRESSO analysis [24], and then conducted a second round of MR analysis to assess the robustness of our results. Furthermore, multivariable Mendelian randomization analysis (MVMR) was performed to assess the causal effect of sarcopenia traits on GERD after adjusting five confounding factors (BMI, smoking initiation, alcohol intake frequency, coffee intake, and type 2 diabetes).

In general, P < 0.05 was considered statistically significant. If multiple comparisons were conducted, the Bonferroni-corrected P < 0.0167 (0.05/3) was considered statistically significant. All analyses were conducted using the Two-Sample MR [45] and MR-PRESSO [24] packages in R software version 4.2.1.

## Supplementary Material

Supplementary Table 1

Supplementary Table 2

Supplementary Tables 3-6
