# Supplementary material for: A Mendelian randomization analysis identifies causal association between sarcopenia and gastroesophageal reflux disease
Source: Aging (Albany NY). 2024 Mar 5;16(5):4723–35. doi: 10.18632/aging.205627 (PMC10968686; doi:10.18632/aging.205627)
Supplement: Supplementary Table 1 [file aging-16-205627-s001.pdf]

SUPPLEMENTARY TABLES

Supplementary Table 1. Data sources used in this Mendelian randomization study.

Traits	Sample size	SNPs (n)	Population	Consortium or study	PMID
**Main variables**					
Low hand grip strength	256523	9336415	European	UK Biobank	33510174
Usual walking pace	459915	9851867	European	UK Biobank	
Appendicular lean mass	450243	18071518	European	UK Biobank	33097823
Gastroesophageal reflux disease	602604	2320781	European	Ong JS et.al.	34187846
**Confounding variables**					
Body mass index	681275	2336260	European	GIANT	30643251
Smoking initiation	607291	11802365	European	GSCAN	30643251
Alcohol intake frequency	462346	9851867	European	MRC-IEU	
Coffee intake	428860	9851867	European	MRC-IEU	
Type 2 diabetes	655666	5030727	European	Xue Angli, et al.	30054458

SNP, single nucleotide polymorphism; GIANT, Genetic Investigation of Anthropometric Traits; GSCAN, GWAS and Sequencing Consortium of Alcohol and Nicotine use; MRC-IEU, Medical Research Council Integrative Epidemiology Unit.
