# Supplementary material for: A Mendelian randomization analysis identifies causal association between sarcopenia and gastroesophageal reflux disease
Source: Aging (Albany NY). 2024 Mar 5;16(5):4723–35. doi: 10.18632/aging.205627 (PMC10968686; doi:10.18632/aging.205627)
Supplement: Supplementary Tables 3-6 [file aging-16-205627-s003.pdf]

Supplementary Table 3. Heterogeneity and pleiotropy assessment for instrumental variables used in this study.

Outcome	Exposure	Q	Q_df	P	Egger_intercept	SE	P
**Forward**							
GERD	Low hand grip strength	55.63709	9	0.000000009207236	-0.05234742	0.01565069	0.01015941
GERD	Usual walking pace	178.5129	45	8.23772E-18	0.006452842	0.0070507	0.3650722
GERD	ALM	1087.236	403	3.069009E-64	-0.00203148	0.001100489	0.06563016
**Reverse**							
Low hand grip strength	GERD	165.9487	76	0.00000001192038	-0.0135289	0.007493255	0.07501297
Usual walking pace	GERD	279.2272	76	5.277281E-25	0.001943671	0.001693476	0.2547235
ALM	GERD	747.4698	76	6.02901E-111	0.002187529	0.004155044	0.6001114

SE, standard error; GERD, gastroesophageal reflux disease; ALM, appendicular lean mass.

Supplementary Table 4. Outliers identified by Mendelian randomization pleiotropy residual sum and outlier method.

Outcome	Exposure	Outliers SNPs
Forward		
GERD	Low hand grip strength	"rs13107325" "rs2899611"
GERD	Usual walking pace	"rs10883618" "rs2280406" "rs57800857"
GERD	ALM	"rs10471339" "rs1047891" "rs12962050" "rs17718736" "rs1823217" "rs2112617" "rs34522021" "rs3764002" "rs55872725" "rs6142059" "rs6425817" "rs7144307" "rs7543202" "rs7902" "rs9375188" "rs9388490" "rs990315"
**Reverse**		
Low hand grip strength	GERD	"rs13107325"
Usual walking pace	GERD	"rs13107325" "rs3766823" "rs903959"
ALM	GERD	"rs12967855" "rs12997558" "rs1510719" "rs1716171" "rs2145318" "rs2240326" "rs2744961" "rs2782641" "rs3828917" "rs773109" "rs903678" "rs9372625" "rs957345" "rs9940128"

SNP, single nucleotide polymorphism; GERD, gastroesophageal reflux disease; ALM, appendicular lean mass.

Supplementary Table 5. Causal effects for sarcopenia traits on gastroesophageal reflux disease.

Exposure	SNPs (n)	Method	P	OR	95% C.I.	
					Lower	Upper
Low hand grip strength	10	Inverse variance weighted	**0.0098976798307882**	1.23576651056185	1.05214733391896	1.4514306308586
		MR Egger	**0.00410803139665251**	3.4276924911422	1.86636179921092	6.29517589719207
		Weighted median	**0.00921033288076617**	1.15505995600417	1.03629999116048	1.28742981119813
		Weighted mode	0.232575696532655	1.12206476661823	0.940640643005696	1.33848069382051
		Maximum likelihood	**3.0150996054843E-09**	1.24714316565729	1.15935896360659	1.34157420132172
Usual walking pace	46	Inverse variance weighted	**4.18450775147641E-34**	0.118128751992712	0.0837582490112389	0.166603316235552
		MR Egger	**0.000742079157846699**	0.0588709788375193	0.0127384237381341	0.27207386255431
		Weighted median	**8.66519813610834E-29**	0.146667181541599	0.1046076485531	0.205637565119695
		Weighted mode	**2.16063180148976E-06**	0.13486784007097	0.0654527336837425	0.27790029937171
		Maximum likelihood	**9.56609371241157E-102**	0.105980651398351	0.0863004761339655	0.130148742787738
ALM	404	Inverse variance weighted	**1.27748918189711E-12**	0.861157597594515	0.82632999065072	0.897453095355725
		MR Egger	0.231967632041971	0.939853395616918	0.849087050200934	1.04032255001842
		Weighted median	**2.11837177487596E-07**	0.883169358043768	0.842679048122258	0.925605207255941
		Weighted mode	**0.00345632900771261**	0.847091089672808	0.758412805087719	0.946138184098923
		Maximum likelihood	**2.45681711955831E-31**	0.859296871393767	0.837640995714832	0.881512625294781
**After removing outlier SNPs by MR-PRESSO**						
Low hand grip strength	8	Inverse variance weighted	**0.000299999027730516**	1.19547392751544	1.08518178921398	1.31697557549723
		MR Egger	**0.446084442517803**	1.47056768674622	0.581854575346502	3.71668353731562
		Weighted median	**0.00312452990659849**	1.17333163838139	1.05530624889625	1.30455698056053
		Weighted mode	0.614410964795861	1.05648009604846	0.861259106811536	1.29595168807987
		Maximum likelihood	**0.0000022581238297673**	1.20353178927829	1.11457587476416	1.29958740414142
Usual walking pace	43	Inverse variance weighted	**3.67601209664597E-41**	0.129074899077959	0.0957508860957471	0.173996609862446
		MR Egger	**0.000428292378106563**	0.0799112393395582	0.0219459431461375	0.29097888982311
		Weighted median	**1.19258854978364E-28**	0.146942079691838	0.104747470008989	0.206133616232541
		Weighted mode	**0.0000252349962965257**	0.142556774361654	0.0636237985190927	0.319415602171269
		Maximum likelihood	**6.9028271990995E-89**	0.113461914970098	0.0916585966578218	0.140451704674698
ALM	387	Inverse variance weighted	**3.58508881349654E-13**	0.874152214784475	0.843023473681725	0.906430388320477
		MR Egger	0.132753021742632	0.93327126262351	0.853076325479394	1.02100506557771
		Weighted median	**1.61256951347889E-07**	0.885749284588662	0.846446872887703	0.926876594715014
		Weighted mode	**0.00443462754602257**	0.846700681317431	0.755513740699782	0.948893455040781
		Maximum likelihood	**1.12331785710697E-24**	0.872868350563424	0.850476939120862	0.895849284523679

SNP, single nucleotide polymorphism; GERD, gastroesophageal reflux disease; ALM, appendicular lean mass; OR, odds ratio; C.I., confidence interval; P-value in bold means statistical significance.

Supplementary Table 6. Causal effects for gastroesophageal reflux disease on sarcopenia traits.

Outcome	SNPs (n)	Method	P	OR	95% C.I.	
					Lower	Upper
Low hand grip strength	77	Inverse variance weighted	**8.80150028146095E-09**	1.26940957105684	1.17031147327465	1.37689896740211
		MR Egger	**0.00618789008392623**	1.90745953090385	1.21713730718876	2.98931093521374
		Weighted median	**0.0000801291239117278**	1.20021701682021	1.09615301043013	1.31416040804335
		Weighted mode	0.305424035116786	1.14554375941081	0.884946071084934	1.48288189258382
		Maximum likelihood	**1.36832112127314E-17**	1.28284540266006	1.21154892288525	1.35833749346821
Usual walking pace	77	Inverse variance weighted	**7.4483023541907E-45**	0.878184440721318	0.862416751925898	0.894240412425661
		MR Egger	**0.000501993924909125**	0.828298767112685	0.748356458717957	0.916780819632058
		Weighted median	**2.32154287362019E-28**	0.901389039206421	0.884934410391695	0.918149628334422
		Weighted mode	**0.00247715257266538**	0.920481768930913	0.873945799157094	0.969495691553614
		Maximum likelihood	**1.24521491085552E-122**	0.876693191219204	0.86714369965739	0.886347847345006
ALM	77	Inverse variance weighted	**0.000364982691626887**	0.922715360459826	0.882791195142887	0.964445093146517
		MR Egger	0.253339336013414	0.863942800587718	0.673501693029472	1.10823353588019
		Weighted median	**7.13024487912684E-08**	0.925016876099804	0.899157033743548	0.951620449997486
		Weighted mode	0.0719680196948573	0.937249310272339	0.874227013025839	1.00481483243759
		Maximum likelihood	**2.85214320625278E-29**	0.9089103587653	0.893887100616319	0.924186107732478
**After removing outlier SNPs by MR-PRESSO**						
Low hand grip strength	76	Inverse variance weighted	**1.58060953344489E-08**	1.24227498918164	1.15225407150452	1.33932887451741
		MR Egger	0.224507893710291	1.33837927006632	0.839520775403998	2.13366854403484
		Weighted median	**0.000105472011109993**	1.19569210791802	1.09241028167981	1.30873870460006
		Weighted mode	0.301411865182194	1.14435336992721	0.887684552523829	1.47523648073013
		Maximum likelihood	**1.58125003917004E-14**	1.25255268865902	1.18260627022782	1.32663615724336
Usual walking pace	74	Inverse variance weighted	**6.15504506438511E-52**	0.881942110402663	0.867736482163386	0.896380297578692
		MR Egger	0.0913440125607537	0.916420814503126	0.829238429329959	1.01276916210114
		Weighted median	**1.07814646013318E-31**	0.901760933164237	0.886293130995186	0.917498683159321
		Weighted mode	**0.00210353966790448**	0.921409306152112	0.876203569840043	0.968947329920952
		Maximum likelihood	**1.4122513219131E-111**	0.882314132757001	0.872720020592483	0.892013716305299
ALM	65	Inverse variance weighted	**2.89096890446525E-09**	0.921746624157108	0.89728412315994	0.946876042064529
		MR Egger	0.136217630663863	0.882298846120428	0.749880625902853	1.0381002348583
		Weighted median	**1.16537543713167E-07**	0.924815257889613	0.898460522775266	0.95194306209864
		Weighted mode	0.11445496470313	0.939689151024915	0.870755639312196	1.01407979539635
		Maximum likelihood	**1.40408576841019E-22**	0.920502101144467	0.905343332457902	0.935914683229611

SNP, single nucleotide polymorphism; GERD, gastroesophageal reflux disease; ALM, appendicular lean mass; OR, odds ratio; C.I., confidence interval; P-value in bold means statistical significance.
